# Functional recovery after percutaneous revascularization of coronary chronic total occlusions: insights from cardiac magnetic resonance tissue tracking

**DOI:** 10.1007/s10554-021-02355-4

**Published:** 2021-08-02

**Authors:** Henk Everaars, Stefan P. Schumacher, Wijnand J. Stuijfzand, Martijn van Basten Batenburg, Jennifer Huynh, Pepijn A. van Diemen, Michiel J. Bom, Ruben W. de Winter, Peter M. van de Ven, Ramon B. van Loon, Albert C. van Rossum, Maksymilian P. Opolski, Alexander Nap, Paul Knaapen

**Affiliations:** 1 Department of Cardiology, Amsterdam UMC, Vrije Universiteit, ZH 5F003, De Boelelaan 1117, 1081 HV Amsterdam, The Netherlands; 2 Department of Epidemiology and Biostatistics, Amsterdam UMC, Vrije Universiteit, Amsterdam, The Netherlands; 3 Department of Interventional Cardiology and Angiology, National Institute of Cardiology, Warsaw, Poland

**Keywords:** Coronary occlusion, Magnetic resonance imaging, Ventricular function, left, Percutaneous coronary intervention

## Abstract

To evaluate the effect of percutaneous coronary intervention (PCI) of coronary chronic total occlusions (CTOs) on left ventricular (LV) strain assessed using cardiac magnetic resonance (CMR) tissue tracking. In 150 patients with a CTO, longitudinal (LS), radial (RS) and circumferential shortening (CS) were determined using CMR tissue tracking before and 3 months after successful PCI. In patients with impaired LV strain at baseline, global LS (10.9 ± 2.4% vs 11.6 ± 2.8%; *P* = 0.006), CS (11.3 ± 2.9% vs 12.0 ± 3.5%; *P* = 0.002) and RS (15.8 ± 4.9% vs 17.4 ± 6.6%; *P* = 0.001) improved after revascularization of the CTO, albeit to a small, clinically irrelevant, extent. Strain improvement was inversely related to the extent of scar, even after correcting for baseline strain (B =  − 0.05; *P* = 0.008 for GLS, B =  − 0.06; *P* = 0.016 for GCS, B =  − 0.13; *P* = 0.017 for GRS). In the vascular territory of the CTO, dysfunctional segments showed minor improvement in both CS (10.8 [6.9 to 13.3] % vs 11.9 [8.1 to 15.0] %; *P* < 0.001) and RS (14.2 [8.4 to 18.7] % vs 16.0 [9.9 to 21.8] %; *P* < 0.001) after PCI. Percutaneous revascularization of CTOs does not lead to a clinically relevant improvement of LV function, even in the subgroup of patients and segments most likely to benefit from revascularization (i.e. LV dysfunction at baseline and no or limited myocardial scar).

**Supplementary Information:**

The online version contains supplementary material available at 10.1007/s10554-021-02355-4.

## Introduction

Coronary chronic total occlusions (CTOs) are present in approximately 1 in 4 patients with obstructive coronary artery disease (CAD) on invasive coronary angiography [1]. The presence of a CTO confers unfavorable prognosis, with higher rate of major adverse cardiovascular events including death [2]. Contemporary guidelines consider the treatment of CTO lesions analogous to that of non-CTO lesions, indicating that revascularization is recommended for relieving symptoms in patients with refractory angina refractory and for improving prognosis in patients with a large area of viable myocardium at risk [3]. Percutaneous coronary intervention (PCI) of CTO lesions however comes at the expense of higher contrast use, longer fluoroscopy time and increased complication rates in comparison with non-CTO lesions [4]. In addition, CTO-PCI did not improve outcome compared with conservative treatment in 3 recent randomized trials [5–7]. The benefit of CTO-PCI is therefore controversial and careful selection of patients is required before attempting revascularization. Functional recovery of hibernating, viable myocardium is one of the potential benefits of CTO-PCI. Cardiac magnetic resonance imaging (CMR) is considered the gold standard for quantifying cardiac function and has been extensively used to study functional recovery after CTO-PCI, with conflicting results [8–19]. Furthermore, no differences in regional function in the CTO territory between patients treated with CTO-PCI versus patients receiving optimal medical therapy only was observed [11]. Importantly, prior studies used left ventricular (LV) ejection fraction and analysis of wall thickening to measure global and regional function, respectively. Assessment of myocardial strain using CMR tissue tracking has been proposed as a more sensitive method to measure LV dysfunction [20]. Myocardial strain provides prognostic information incremental to LV ejection fraction and is superior to wall thickening in quantifying regional myocardial function [20, 21]. Therefore, the aim of the present study was to evaluate the effect of CTO-PCI on global and regional myocardial strain assessed using CMR tissue tracking.

## Materials and methods

### Study population and design

Consecutive patients with a CTO of a native coronary artery referred to the Amsterdam University Medical Center were prospectively enrolled between 2013 and 2018. CTO was defined as a vessel with Thrombolysis In Myocardial Infarction (TIMI) flow grade 0 or 1 with an estimated duration of ≥ 3 months. Baseline CMR was performed prior to CTO-PCI in all patients and follow-up CMR was scheduled 3 months after successful percutaneous revascularization. Exclusion criteria were recent myocardial infarction, concomitant non-ischemic cardiomyopathy and non-diagnostic baseline or follow-up CMR cine images. Since myocardial strain values are dependent on acquisition method and post-processing software, 100 healthy volunteers without a history of cardiovascular disease underwent CMR to calculate normal values. The study was approved by the Institutional Review Board of the Amsterdam University Medical Center, location VUmc. All subjects provided written informed consent.

### CMR image acquisition

CMR was performed in all subjects on a 1.5-T clinical scanner (Magnetom Avanto, Siemens Healthineers) using identical imaging parameters. Cine images were obtained using a balanced steady-state free-precession sequence in the 2-, 3- and 4-chamber long-axis views and multiple short-axis views covering the entire LV from base to apex. Typical imaging parameters were: echo time, 1.5 ms; repetition time, 3.2 ms; α, 60 to 80°; spatial resolution, 1.6 × 1.6 mm; slice thickness, 5 mm; gap, 5 mm; temporal resolution, 30 to 50 ms. For assessment of myocardial scar, late gadolinium enhancement (LGE) images were obtained 10 to 15 min after administration of 0.2 mmol/kg of a gadolinium-based contrast agent (Dotarem®, Guerbet) using a segmented inversion recovery gradient-echo pulse sequence. Slice positions of the LGE images were identical to those of the cine images. Typical imaging parameters were: echo time, 4.4 ms; repetition time, 9.6 ms; α, 25°; spatial resolution, 1.6 × 1.6 mm; slice thickness, 5 mm; inversion time, 250 to 350 ms.

### CMR image analysis

CMR analysis was performed by a single observer (H.E.) blinded to all clinical data, angiographic data and timing of imaging. Conventional analysis of cine and LGE images was performed using QMass software (version 7.6, Medis Medical Imaging Systems). LV ejection fraction, mass and volumes were calculated from the short-axis cine images. Infarct size was determined from the LGE images using the full-width-at-half maximum method, followed by manual correction [22]. Analysis of myocardial strain was performed using CMR^42^ software (version 5.11, Circle Cardiovascular Imaging Inc.). Endo- and epicardial contours were manually drawn on the end-diastolic and end-systolic phases and reference points for segmentation were manually defined at the anterior- and inferior RV insertion points. In line with prior reports, longitudinal and circumferential strain were expressed as absolute, positive values and the term shortening instead of strain was used [23]. Global longitudinal shortening (GLS) was calculated from the 3 long-axis cine views. Global circumferential and radial shortening (GCS and GRS, respectively) were calculated from the short-axis cine views. For regional analysis, the LV was divided into 16 segments (true apex not included) according to the segmentation model of the American Heart Association (AHA) [24]. A single observer (S.S) assessed coronary dominance using the invasive coronary angiogram. Segments were subsequently assigned to coronary arteries by H.E. accounting for dominance. In a right dominant system, the standard segmentation model of the AHA was used [24]. In a co-dominant system, segments 4 and 10 were transferred from the RCA to the Cx. In a left dominant system, segments 3, 4, 9, 10 and 15 were all transferred from the RCA tot the Cx as the RCA does not supply the left ventricle. Remote myocardium was defined as the myocardial segments opposite to the vascular territory of the CTO. Remote myocardium was only included in analysis if the myocardial segments showed no hyperenhancement and the supplying coronary vessel had < 50% diameter stenosis. Circumferential and radial shortening (CS and RS, respectively) as well as percentage of hyperenhancement were calculated for each segment. Segments with > 50% hyperenhancement were considered non-viable. Cut-off values for global strain were determined by calculating the fifth percentile of GLS, GCS and GRS in the cohort of healthy volunteers. Given that strain values are independent of initial segment length, impaired regional strain was defined using the same cut-off parameters as used for global strain. The percentage of LV that was dysfunctional but viable was calculated for every patient by dividing the number of segments with ≤ 50% LGE and impaired strain by 16 and multiplying this value with 100. CMR strain analysis was repeated by the first observer (H.E) as well as a second observer (M.B.) in 50 randomly selected scans to assess intra- and interobserver variability, respectively.

### Statistical analysis

Pearson’s and spearman’s correlations were used to quantify association between normally and non-normally distributed continuous data, respectively. Reproducibility of CMR strain measurements was evaluated by intraclass correlation coefficients (ICCs) and Bland–Altman analysis. ICCs for absolute agreement of single measures were estimated using a two-way random effects model. Paired samples T-tests were used to compare global strain and scar size before and after CTO-PCI. Association between extent of scar and global strain improvement was tested using analysis of covariance with global strain improvement as dependent variable and correcting for global strain at baseline. On a segmental level, means of strain before and after CTO-PCI were compared using a linear mixed model with a fixed effect for imaging timing and random effects for patient and segment nested within patient. All statistical tests were two tailed and a p value of < 0.05 was considered statistically significant. Statistical analysis was done with SPSS (version 26 for Windows, IBM).

## Results

### Characteristics of the study population

**Fig. 1:**
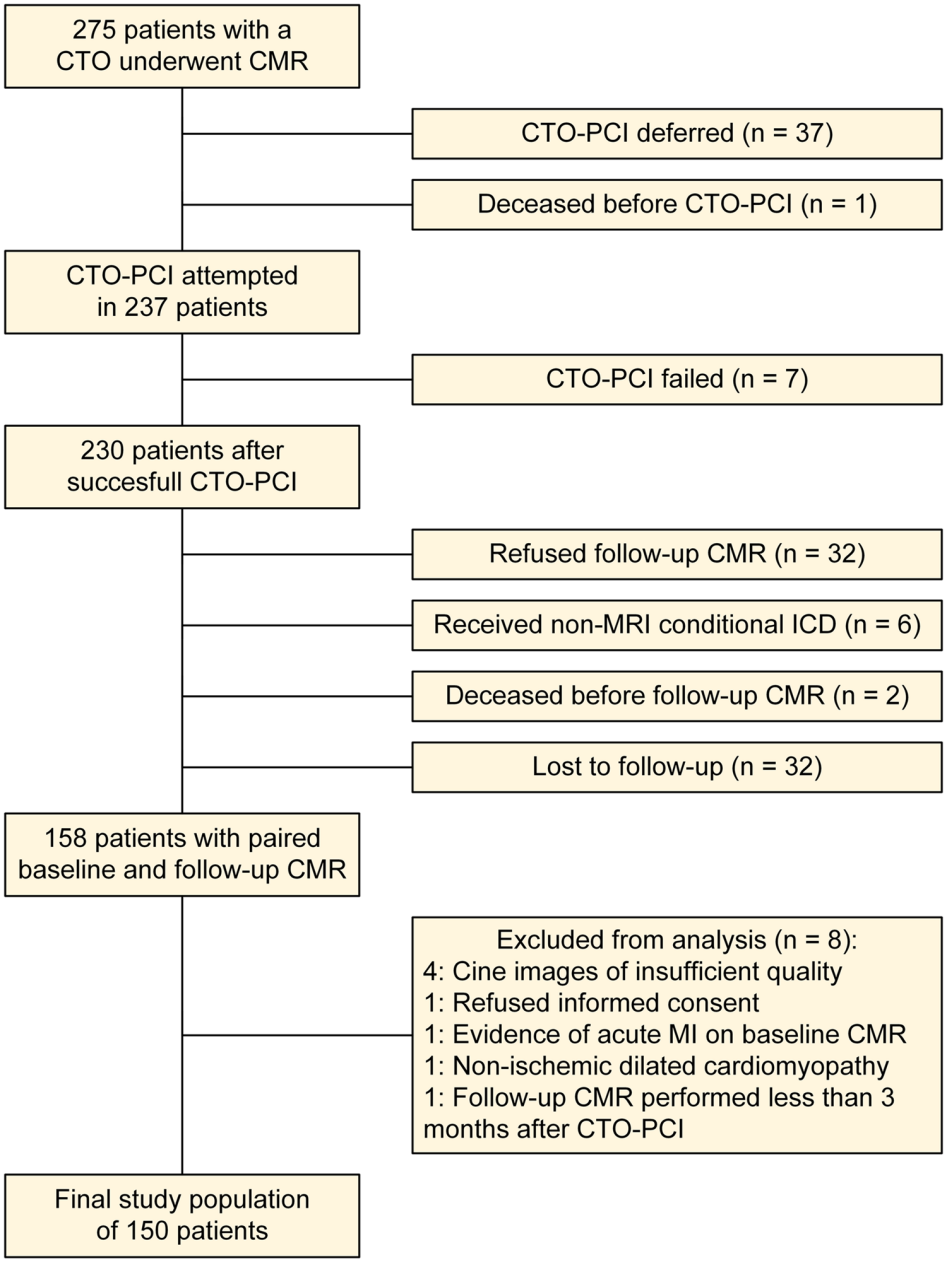
Flow chart of study population. *CMR* cardiac magnetic resonance imaging;* CTO-PCI* percutaneous coronary intervention of a coronary chronic total occlusion;* ICD* implantable cardioverter-defibrillator

Figure 1 displays the flowchart of the study population. A total of 275 patients underwent CMR, CTO-PCI was attempted in 237 (86%) patients and paired CMR images were available in 158 (57%) patients. Eight patients were excluded post-hoc (reasons presented in Fig. 1) resulting in a final sample size of 150 patients. Table 1 lists the baseline characteristics of the study cohort and Table 2 lists angiographic characteristics. Median time between PCI and follow-up CMR was 101 [94 to 117] days.

**Table 1:** Baseline characteristics of the study cohort

Variables	N = 150
Age (years)	63 ± 11
Male	124 (83)
Body mass index (kg/m^2^)	27 ± 4
Risk factors	
Family history of CAD	67 (45)
Hypertension	75 (50)
Dyslipidemia	66 (44)
Diabetes mellitus	37 (25)
Smoking	111 (74)
Peripheral artery disease	22 (15)
Cardiac history	
Prior documented MI	73 (49)
Prior documented MI in CTO territory	30 (20)
Prior PCI	104 (69)
Prior PCI in CTO vessel	28 (19)
Prior CABG	17 (11)
Prior CABG on CTO vessel	13 (9)
CCS class	
No angina	78 (52)
I	6 (4)
II	45 (30)
III	18 (12)
IV	3 (2)
NYHA class	
I	84 (56)
II	36 (24)
III	29 (19)
IV	1 (1)
Medication	
Aspirin	136 (91)
P2Y12 inhibitor	101 (67)
ACE inhibitor or ATII antagonist	79 (53)
Beta-blocker	115 (77)
Calcium channel blockers	32 (21)
Long-acting nitrates	119 (79)
Statin	124 (83)

Data are mean ± standard deviation or absolute number (%). CABG = coronary artery bypass grafting; CAD = coronary artery disease; CCS = Canadian Cardiovascular Society; CTO = chronic total occlusion; MI = myocardial infarction; NYHA = New York Heart Association; PCI = percutaneous coronary intervention

**Table 2:** Angiographic characteristics

Variables	All patients (n = 150)	with preserved strain (n = 81)	with impaired strain (n = 51)	P value
CTO vessel				0.38
RCA	105 (70)	55 (68)	37 (73)	
LAD	32 (21)	20 (25)	8 (16)	
LCx	13 (9)	6 (7)	6 (12)	
CTO characteristics				
Blunt stump	42 (28)	18 (22)	18 (35)	0.28
Occlusion length ≥ 20 mm	82 (55)	31 (38)	23 (45)	0.44
Severe calcification	88 (59)	40 (49)	36 (71)	0.11
Bending > 45°	57 (38)	36 (44)	15 (29)	0.21
Ostial location	13 (9)	9 (11)	2 (4)	0.15
Previous failed PCI attempt	25 (17)	15 (19)	7 (14)	0.47
J-CTO score				0.27
0–1	56 (37)	35 (43)	17 (33)	
2	49 (33)	23 (28)	19 (37)	
≥ 3	45 (30)	23 (28)	15 (29)	
Collateral collection score				0.52
0	6 (4)	4 (5)	3 (6)	
1	41 (27)	22 (27)	17 (33)	
2	103 (69)	55 (68)	31 (61)	
Rentrop grade				0.55
0–1	1 (1)	1 (1)	0 (0)	
2	20 (13)	10 (12)	10 (20)	
3	129 (86)	70 (86)	41 (80)	
Extent of CAD				0.02
1-vessel disease	101 (67)	61 (75)	27 (53)	
2-vessel disease	42 (28)	18 (22)	19 (37)	
3-vessel disease	7 (5)	2 (3)	5 (10)	
Successful PCI strategy				0.61
AWE	65 (43)	35 (43)	24 (47)	
ADR	31 (21)	18 (22)	7 (14)	
RWE	19 (13)	9 (11)	8 (16)	
RDR	35 (23)	19 (24)	12 (24)	
Number of implanted stents	2 [2, 3]	2 [1–3]	2 [2, 3]	0.10
Total stent length (mm)	83 ± 39	82 ± 40	87 ± 37	0.26
Contrast volume (mL)	300 [200–400]	300 [200–400]	310 [200–400]	0.57
Fluoroscopy time (min)	33 [18–53]	30 [17–51]	31 [21–53]	0.90
Periprocedural adverse events				
Myocardial infarction	7 (5)	4 (5)	1 (2)	0.38
Coronary perforation	13 (9)	6 (7)	5 (10)	0.63
Tamponade	3 (2)	2 (3)	1 (2)	0.85
Emergency CABG	0 (0)	0 (0)	0 (0)	–
Stroke or TIA	1 (1)	1 (1)	0 (0)	0.43
Death	0 (0)	0 (0)	0 (0)	–

Data are mean ± standard deviation, median [interquartile range] or absolute number (%)

*ADR* antegrade dissection and re-entry, *AWE* antegrade wire escalation, *J-CTO score* Japanese chronic total occlusion score, *RDR* retrograde dissection and re-entry, *RWE* retrograde wire escalation; other abbreviations as in Table 1

### CMR assessment of myocardial scar

Although only 30 (20%) patients had a documented prior myocardial infarction related to the CTO vessel, CMR revealed hyperenhancement in the vascular territory of the CTO in 112 (75%) patients.

Median scar size was 4.4 [1.0 to 10.4] % before revascularization and increased to 5.0 [2.1 to 14.4] % after (*P* < 0.001).

### Reproducibility of strain measurements

Global and regional strain measurements had excellent intra- and interobserver reproducibility (Table 3). Bland–Altman plots demonstrated no significant bias and narrow limits of agreement (Supplementary Figs. 1 and 2).

**Table 3:** Intraclass correlation coefficients of strain measurements

	Intraobserver	Interobserver
Global		
GLS	0.98 (0.97 to 0.99)	0.97 (0.95 to 0.99)
GCS	0.99 (0.99 to 1.00)	0.99 (0.99 to 1.00)
GRS	0.99 (0.99 to 1.00)	0.99 (0.99 to 1.00)
Regional		
CS	0.95 (0.94 to 0.96)	0.93 (0.92 to 0.94)
RS	0.95 (0.95 to 0.96)	0.93 (0.92 to 0.94)

Data are intraclass correlation coefficients (95% confidence interval)

*GLS* global longitudinal shortening, *GCS* global circumferential shortening, *GRS* global radial shortening, *CS* circumferential shortening, *RS* radial shortening

### Strain in healthy volunteers

In the cohort of healthy volunteers, GLS ranged from 13.3% to 21.7% with a mean 17.6 ± 1.8%, GCS from 13.6% to 23.3% with a mean of 18.5 ± 2.2% and GRS from 20.8% to 46.1% with a mean of 31.3 ± 6.1% (data presented in Supplementary Table 1). Impaired global and radial strain were defined as LS < 14.4%, CS < 15.0% and RS < 22.1%.

### Global LV function

**Fig. 2:**
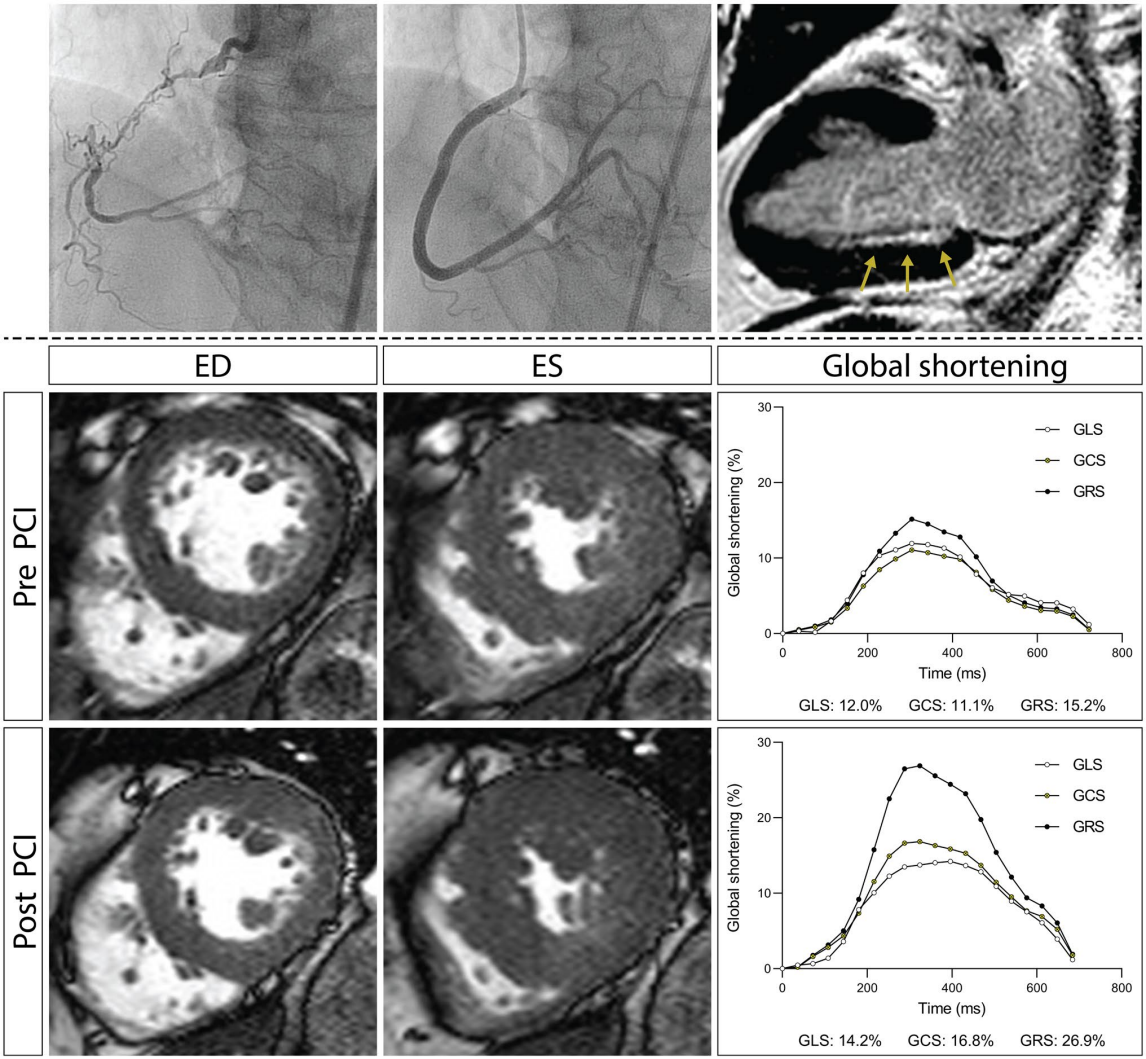
Case example of a patient with a CTO of the RCA. The RCA is proximally occluded and the distal coronary segments are filled through collaterals (top row, left). CMR demonstrates subendocardial hyperenhancement (top row, right, arrows) and regional wall motion abnormalities (middle row, left and center) of the inferior wall. Global longitudinal, circumferential and radial strain are all impaired (middle row, right). Successful restoration of coronary patency (top, middle) results in marked improvements of global myocardial shortening (bottom row). *CMR* cardiac magnetic resonance imaging, *CTO* coronary chronic total occlusion, *ED* end-diastole, *ES* end-systole, *GCS* global circumferential shortening, *GLS* global longitudinal shortening, *GRS* global radial shortening, *PCI* percutaneous coronary intervention, *RCA* right coronary artery

**Fig. 3:**
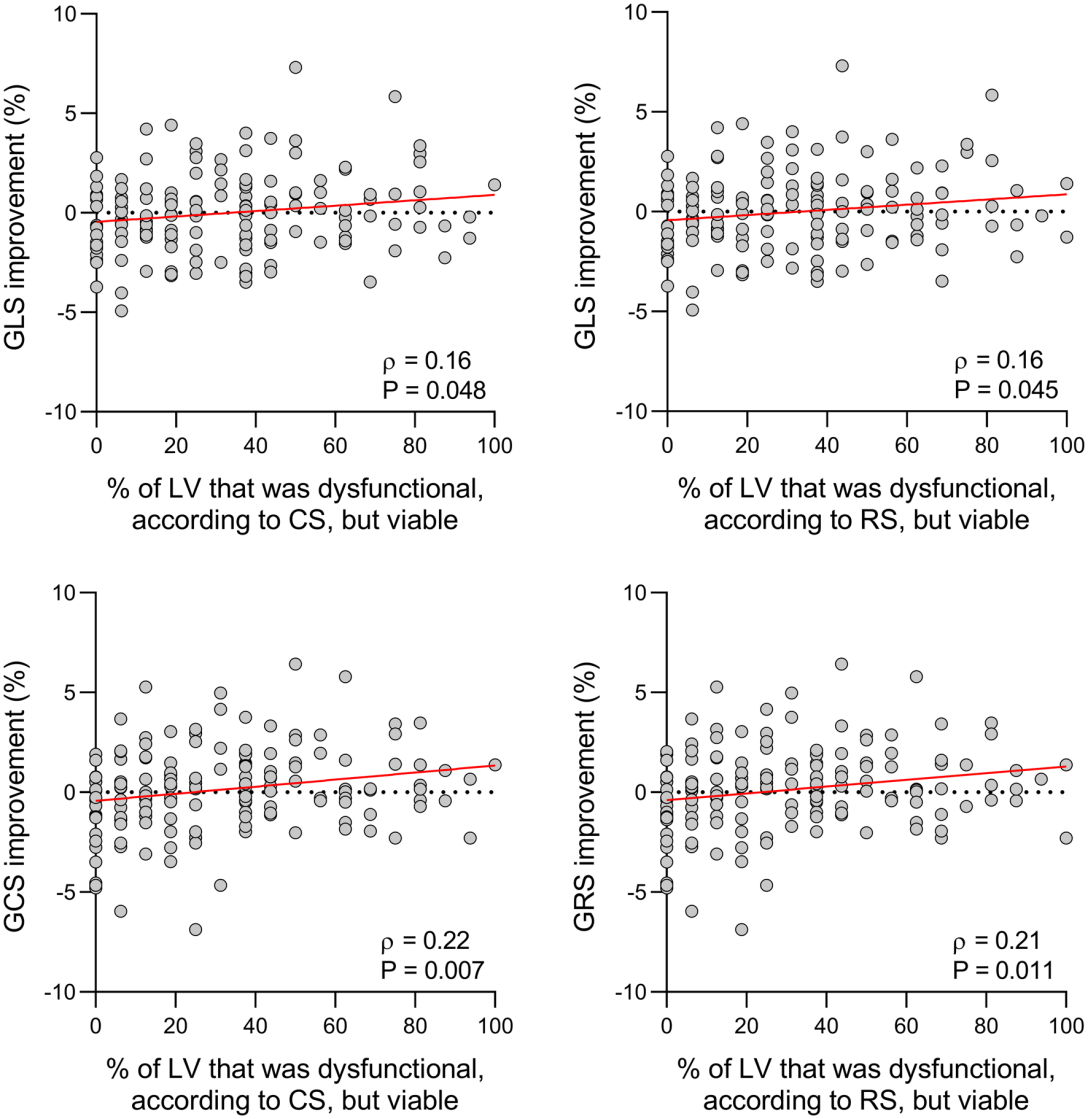
Relationship between global strain improvement and extent of viability. Scatterplots demonstrating the relationship of improvement in GLS (top), GCS (bottom, left) and GRS (bottom, right) with the percentage of LV that was dysfunctional but viable. *CS* circumferential shortening, *RS* radial shortening; other abbreviations as in Fig. 2

GLS, GCS and GRS correlated strongly with LV ejection fraction (*r* = 0.86; *P* < 0.001 for GLS, *r* = 0.93; *P* < 0.001 for GCS, *r* = 0.90; *P* < 0.001 for GRS) and inversely with the extent of myocardial scar (ρ =  − 0.59; *P* < 0.001 for GLS, ρ =  − 0.64; *P* < 0.001 for GCS, ρ =  − 0.63; *P* < 0.001 for GRS) (Supplementary Fig. 3). GLS was impaired in 62 (41%) patients, GCS in 60 (40%) patients and GRS in 58 (39%) patients. A total of 51 (34%) patients had impaired LV strain, defined as the combination of impaired GLS, GCS and GRS. Table 4 presents global LV function before and after PCI. In the overall population, CTO-PCI resulted in a small but significant improvement in LV ejection fraction and a reduction in LV volumes. In contrast, GLS, GCS and GRS did not improve after CTO-PCI. The subgroup of patients with impaired strain at baseline however demonstrated an increase in GLS, GCS and GRS. Figure 2 depicts a case example of a patient with impaired LV function at baseline in whom strain markedly improved after CTO-PCI. Notably, GLS decreased in patients with preserved strain at baseline while no significant changes in GCS, GRS or LV ejection fraction were observed. Figure 3 shows the relationship between global strain improvement and the extent of viability (i.e. the percentage of the LV that was dysfunctional but viable). Strain improvement correlated significantly with the extent of viability, defined by either CS or RS. In addition, the improvements in strain after CTO-PCI were inversely related to the extent of scar, even after correcting for baseline strain (B =  − 0.05; *P* = 0.008 for GLS, B =  − 0.06; *P* = 0.016 for GCS, B =  − 0.13; *P* = 0.017 for GRS).

**Table 4:** Global LV function, mass and infarct size before and after percutaneous revascularization

	All patients (n = 150)			Patients with preserved^a^ LV strain (n = 81)			Patients with impaired^a^ LV strain (n = 51)			ANCOVA P value
	Before PCI	After PCI	P value	Before PCI	After PCI	P value	Before PCI	After PCI	P value	
EDV (mL/m^2^)	99 ± 32	95 ± 30	< 0.001	85 ± 20	83 ± 20	0.04	125 ± 35	118 ± 34	0.002	0.86
ESV (mL/m^2^)	54 ± 29	51 ± 28	< 0.001	38 ± 12	36 ± 12	0.007	82 ± 32	76 ± 33	< 0.001	0.31
SV (mL/m^2^)	45 ± 10	45 ± 10	0.53	47 ± 10	47 ± 10	0.75	43 ± 10	42 ± 9	0.56	0.50
Ejection fraction (%)	48.3 ± 11.8	49.7 ± 11.8	0.001	54.7 ± 6.5	55.1 ± 6.7	0.40	35.9 ± 9.7	37.9 ± 10.7	0.003	0.67
GLS (%)	14.4 ± 3.4	14.5 ± 3.6	0.90	16.6 ± 2.0	16.2 ± 2.2	0.03	10.4 ± 2.4	11.1 ± 2.7	0.020	0.26
GCS (%)	15.9 ± 4.6	16.0 ± 4.5	0.43	18.5 ± 2.7	18.4 ± 2.9	0.44	10.8 ± 2.9	11.5 ± 3.5	0.007	0.93
GRS (%)	26.2 ± 10.4	26.4 ± 10.2	0.69	31.9 ± 7.5	31.4 ± 7.9	0.40	15.1 ± 4.8	16.7 ± 6.4	0.004	0.31
LV mass (g/m^2^)	52.5 ± 12.5	52.5 ± 12.6	0.92	47.2 ± 10.1	47.8 ± 10.4	0.19	61.6 ± 11.4	60.9 ± 12.1	0.50	0.20
Infarct size (%)	4.4 [1.0 to 10.4]	5.0 [2.1 to 14.4]	< 0.001	1.9 [0.2 to 4.8]	2.9 [0.5 to 5.6]	0.01	14.9 [5.3 to 21.6]	18.1 [10.0 to 27.8]	< 0.001	< 0.001

Data are mean ± standard deviation, median [interquartile range] or absolute number (%)

*EDM* end-diastolic mass, *EDV* end-diastolic volume, *ESV* end-systolic volume, *GLS* global longitudinal shortening, *GCS* global circumferential shortening, *GRS* global radial shortening, *LV* left ventricle, *SV* stroke volume

^a^Preserved LV strain is defined as the combination of GLS ≥ 14.4%, GCS ≥ 15.0% and GRS ≥ 22.1%, whereas impaired LV strain is defined as the combination of GLS < 14.4%, GCS < 15.0% and GRS < 22.1%

### Regional LV function

**Fig. 4:**
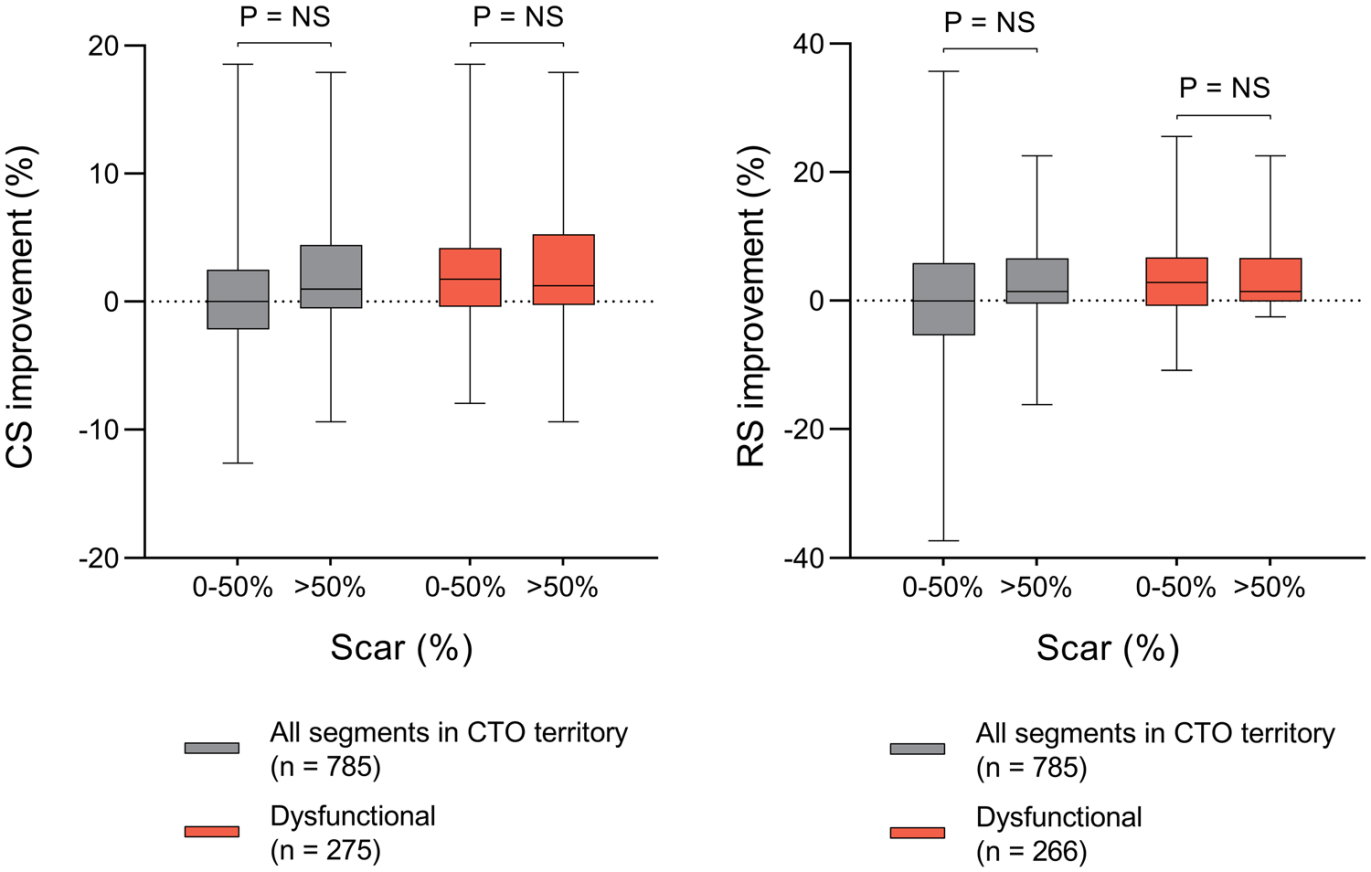
Regional strain improvement. Improvement in CS (top) and RS (bottom) in the vascular territory of the CTO according to baseline strain and extent of hyperenhancement. Abbreviations as in Figs. 2 and 3

Segmental CS and RS correlated inversely with the percentage of scar (ρ =  − 0.48; *P* < 0.001 for CS, ρ =  − 0.47; *P* < 0.001 for RS, Supplementary Fig. 4). Table 5 presents regional strain before and after PCI-CTO stratified according to baseline strain and extent of hyperenhancement. Analysis of all segments in the vascular territory of the CTO revealed a significant increase in CS, but not in RS. In segments with impaired strain at baseline, both CS and RS increased after CTO-PCI. Conversely, CS and RS decreased in segments with preserved strain at baseline. Revascularization had no effect on strain in remote myocardium. Surprisingly, dysfunctional segments with > 50% hyperenhancement demonstrated an increase in CS and RS at follow-up. Although baseline strain was significantly lower in dysfunctional segments with > 50% hyperenhancement compared with dysfunctional segments with ≤ 50% hyperenhancement, the improvement in CS (1.3 [− 0.2 to 5.2] % vs 1.8 [− 0.4 to 4.2] %; *P* = 0.37) and RS (1.5 [− 0.1 to 6.6] % vs 2.8 [− 0.8 to 6.7] %; *P* = 0.97) was similar (Fig. 4).

**Table 5:** Regional LV function before and after percutaneous revascularization

	Before PCI	After PCI	P value
All segments in CTO territory (N = 790)			
CS (%)	15.4 [11.2 to 19.8]	16.0 [11.8 to 19.7]	0.039
RS (%)	23.2 [15.1 to 34.1]	24.3 [15.8 to 33.6]	0.55
Segments in CTO territory with preserved strain (N = 415)^a^			
CS (%)	19.6 [17.4 to 22.7]	19.1 [16.5 to 22.0]	< 0.001
RS (%)	33.5 [27.5 to 42.7]	32.0 [25.5 to 41.1]	< 0.001
Dysfunctional segments in CTO territory with < 50% scar (N = 331)^a^			
CS (%)	11.0 [7.4 to 8.5]	11.9 [8.5 to 15.0]	< 0.001
RS (%)	14.8 [9.7 to 18.7]	16.4 [10.6 to 22.0]	< 0.001
Dysfunctional segments in CTO territory with ≥ 50% scar (N = 32)^a^			
CS (%)	6.1 [0.7 to 9.8]	6.9 [4.1 to 13.5]	< 0.001
RS (%)	7.3 [5.1 to 12.6]	9.0 [4.9 to 18.4]	0.001
Remote segments (N = 710)			
CS (%)	18.7 [14.6 to 22.6]	18.9 [15.2 to 22.1]	0.84
RS (%)	31.0 [21.6 to 42.1]	31.5 [22.4 to 40.9]	0.58

Data are median [inter-quartile range]

^a^Preserved strain is defined as the combination of CS ≥ 15.0% and RS ≥ 22.1%, whereas dysfunctional is defined as the combination of CS < 15.0% and RS < 22.1%

## Discussion

The present study is the largest thus far to investigate functional recovery after CTO-PCI using CMR, which is considered the gold standard for assessment of myocardial function. The main findings of our study can be summarized as follows: (1) CTO-PCI did not improve global longitudinal, circumferential and radial shortening; (2) CTO-PCI resulted in a small but significant improvement of global strain in patients with impaired LV function; (3) This improvement was primarily driven by strain recovery in dysfunctional myocardium in the vascular territory of the CTO.

Previous CMR studies investigating functional improvement after CTO-PCI have shown conflicting results. While some studies reported an increase in LV ejection fraction after CTO-PCI, others found no improvement in LV function [8–19]. Interestingly, mean LV ejection fraction prior to revascularization was well preserved (i.e. > 60%) in studies that failed to demonstrate improvement [8–11]. Conversely, mean baseline LV ejection fraction was impaired in most studies showing an increase in global function [12–19]. Logically, the presence of LV dysfunction appears to be a prerequisite for functional improvement. On a regional level, wall thickening in the vascular territory of the CTO also improves predominantly in segments with hypo- or akinesia at baseline [11]. In addition to LV dysfunction, the presence of viable myocardium has been described as a second prerequisite for functional recovery. Dysfunctional segments with limited or no scar have a high likelihood of improvement, whereas LV function is unlikely to recover in myocardium with transmural infarction [25]. It is therefore imperative to perform both cine and LGE imaging when using CMR to select patients who may benefit from revascularization in terms of functional improvement. Several studies in patients with a CTO reported that LGE imaging aids in predicting functional recovery after PCI [8, 15, 16]. However, Stuijfzand et al. and Fiocchi et al. found no relationship between improvement of regional wall thickening and extent of infarction [13, 17].

In the present study, functional recovery after CTO-PCI was investigated using myocardial strain indices rather than LV ejection fraction and analysis of wall thickening. LV ejection fraction is the cornerstone in the assessment of cardiac function, but is confounded by loading conditions and geometric factors such as LV wall thickness and cavity dimensions [26]. Although myocardial strain is also load dependent, it provides a more accurate assessment of LV function as strain parameters directly quantify myocardial fiber shortening [27]. Myocardial strain imaging is also an auspicious tool to quantify regional function, as conventional analysis of wall thickening suffers from relatively high observer variability due to systolic trabecular infolding [21]. Despite these advantages, myocardial strain imaging using CMR tissue tracking also has shortcomings. Various vendors offer software packages that allow to quantify strain from cine images. In the absence of an industry standard, these software packages use different algorithms with various agreement between the calculated results. In addition, acquisition parameters such as flip angle, spatial and temporal resolution but also the administration of contrast before obtaining the cine images may all influence the calculated strain values to an unknown degree. As a consequence, strain values obtained at one center are not transferable to another center if these factors are not taken into account. In order to usher CMR feature tracking into clinical practice, acquisition protocols and post-processing methods will have to be standardized and harmonized.

Similar to CMR tissue tracking, myocardial strain can also be assessed with echocardiography using a technique referred to as speckle tracking. Speckle tracking echocardiography is more widely available than CMR and has a higher spatial resolution. On the other hand, it has a lower signal-to-noise ratio and may be hampered by poor acoustic windows. Several studies have investigated the effects of CTO-PCI on LV function using speckle tracking echocardiography [28–33]. Although follow-up in these studies ranged from 1 month to 2 years, the majority documented significant improvements in echocardiography derived strain parameters after CTO-PCI. Contrary to these findings, no improvement in LV strain was observed in the overall cohort of present study. Small but statistically significant improvements in global LV function were however noted among patients with LV dysfunction prior to PCI. Conversely, patients with preserved LV function at baseline demonstrated a slight decrease in GLS. These patients have little to gain from PCI in terms of functional recovery, but may still experience loss of contracting cardiomyocytes due to periprocedural injury. This hypothesis is supported by the observation that infarct size increased after CTO-PCI. GCS and GRS remained unaltered in these patients, which is not surprising given that GLS has been documented as being more sensitive to subtle changes in contractility [27]. On a regional level, CS improved in the vascular territory of the CTO but remained unchanged in remote myocardium. Functional recovery after CTO-PCI is consequently a direct result of increased contractility in the vascular territory of the CTO. Functional improvement was most prominent in segments with dysfunction at baseline and was associated with the extent of scar. Surprisingly, strain in dysfunctional segments with > 50% hyperenhancement improved after CTO-PCI similar to the improvement observed in dysfunctional segments with ≤ 50% hyperenhancement.

In summary, LV function did not improve after CTO-PCI. Even in patients with LV dysfunction and myocardial viability at baseline, who are most likely to benefit from revascularization, the functional recovery that could be gained through CTO-PCI was only minor and not clinically relevant. It is therefore unlikely that CTO-PCI will reduce the incidence of heart failure or otherwise significantly improve prognosis for the individual patient through vast improvement of LV function. This should be taken into consideration when scheduling a patient for CTO-PCI, especially given the higher complication rate of CTO-PCI in comparison with regular PCI [4]. CTO-PCI may nevertheless be beneficial to selected patients with borderline LV function who might avoid implantable cardioverter defibrillator and/or cardiac resynchronization therapy through revascularization. In addition, CTO-PCI may halt negative LV remodeling and progression of heart failure, can profoundly reduce ischemic burden and has been shown to improve quality of life [6, 7, 12, 13]. Furthermore, previous studies have demonstrated that LV function may continue to improve up to 3 years after revascularization [10]. To date, randomized trials have consistently failed to show a reduction in hard clinical endpoints, although these trials lacked power and suffered from cross-over [5–7]. Future large-scale studies are therefore warranted to answer the question whether PCI improves cardiovascular outcome in patients with a CTO and these trials should include serial imaging for an extended period.

### Study limitations

The follow-up period of 3 months after successful PCI was arbitrary and longer follow-up may have increased the observed improvement in LV function. Moreover, CTO-PCI was not performed in patients without evidence of viability on baseline CMR. Although such a diagnostic work-up is advocated by current guidelines, the exclusion of patients with predominantly transmural scar in the CTO territory may explain the modest relationship between strain improvement and myocardial scar observed in the present study [3]. Finally, the use of a different contrast agent may have improved the delineation between infarcted myocardium, blood pool and non-infarcted myocardium, and increased slice thickness may have resulted in more optimal cine images.

## Conclusions

Percutaneous revascularization of CTOs does not improve global longitudinal, circumferential or radial shortening. Even in the subgroup of patients and segments most likely to benefit from revascularization (i.e. LV dysfunction at baseline and no or limited myocardial scar), CTO-PCI failed to show a clinically relevant improvement in myocardial contractility. It is therefore unlikely that CTO-PCI will favorably affect patient outcomes through recovery of LV function.

## Supplementary Information

Below is the link to the electronic supplementary material.

Supplementary file1 (DOCX 1029 kb)

## Data Availability

Data will be made available upon reasonable request.

## Abbreviations

CAD: Coronary artery disease
CMR: Cardiac magnetic resonance imaging
CTO: Coronary chronic total cocclusion
(G)CS: (Global) circumferential shortening
GLS: Global longitudinal shortening
(G)RS: (Global) radial shortening
ICC: Intraclass correlation coefficients
LGE: Late gadolinium enhancement
LV: Left ventricular
PCI: Percutaneous coronary intervention
TIMI: Thrombolysis in myocardial infarction

## Code availability

Yes.
