# Supplementary material for: Functional recovery after percutaneous revascularization of coronary chronic total occlusions: insights from cardiac magnetic resonance tissue tracking
Source: Int J Cardiovasc Imaging. 2021 Aug 2;37(10):3057–68. doi: 10.1007/s10554-021-02355-4 (PMC8494704; doi:10.1007/s10554-021-02355-4)
Supplement: Supplementary file 1 — Supplementary file1 (DOCX 1029 kb) [file 10554_2021_2355_MOESM1_ESM.docx]

SUPPLEMENTAL MATERIAL

to manuscript entitled

**Functional recovery after percutaneous revascularization of coronary chronic total occlusions: insights from cardiac magnetic resonance tissue tracking**

# Supplemental Figure 1: Reproducibility of global strain measurements


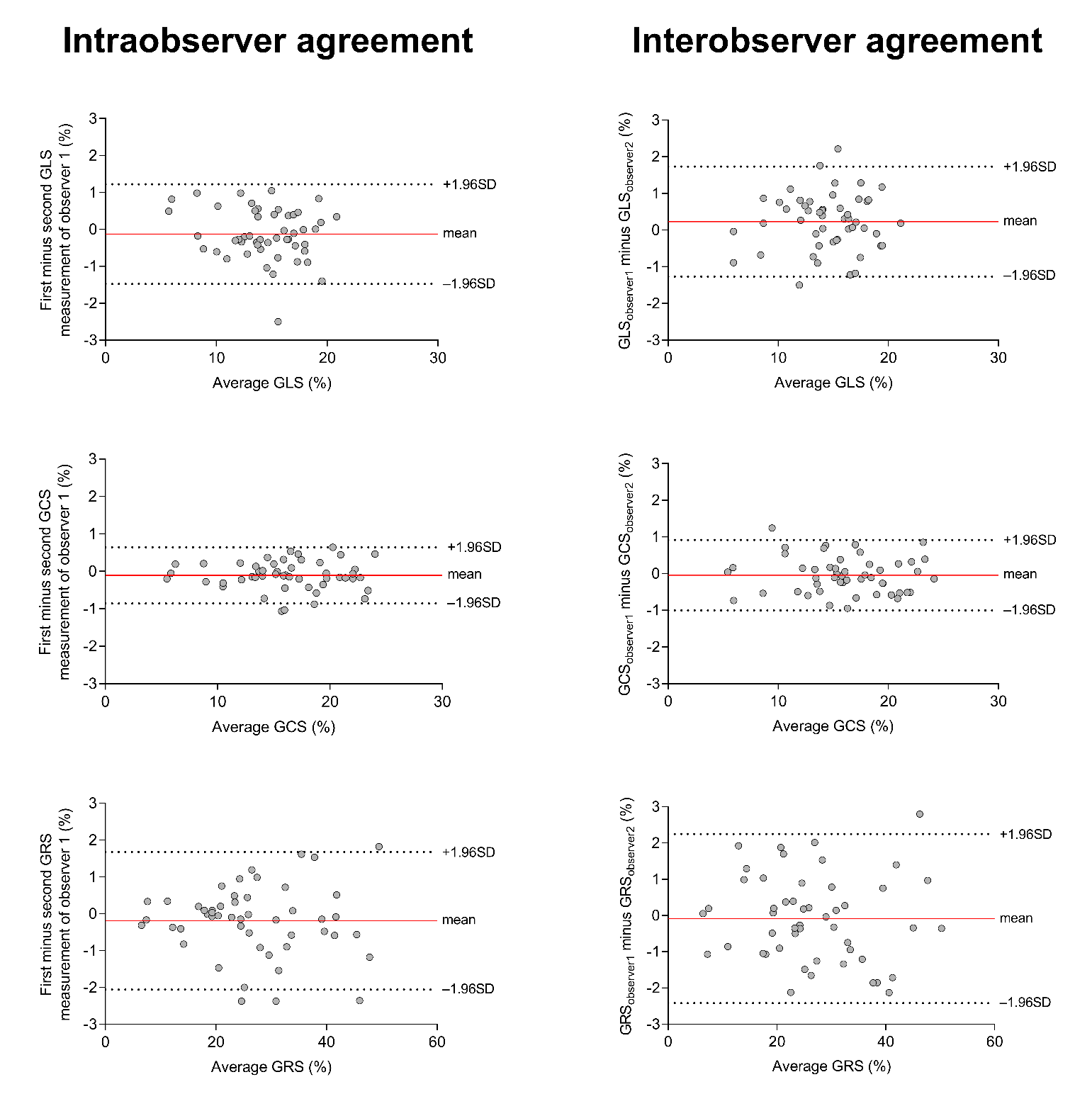


Bland-Altman plots demonstrating the intra- and interobserver agreement of GLS, GCS and GRS. The solid red lines indicate the mean bias and the dashed black lines indicate the limits of agreement. GCS = global circumferential shortening; GLS = global longitudinal shortening; GRS = global radial shortening.

# Supplemental Figure 2: Reproducibility of regional strain measurements


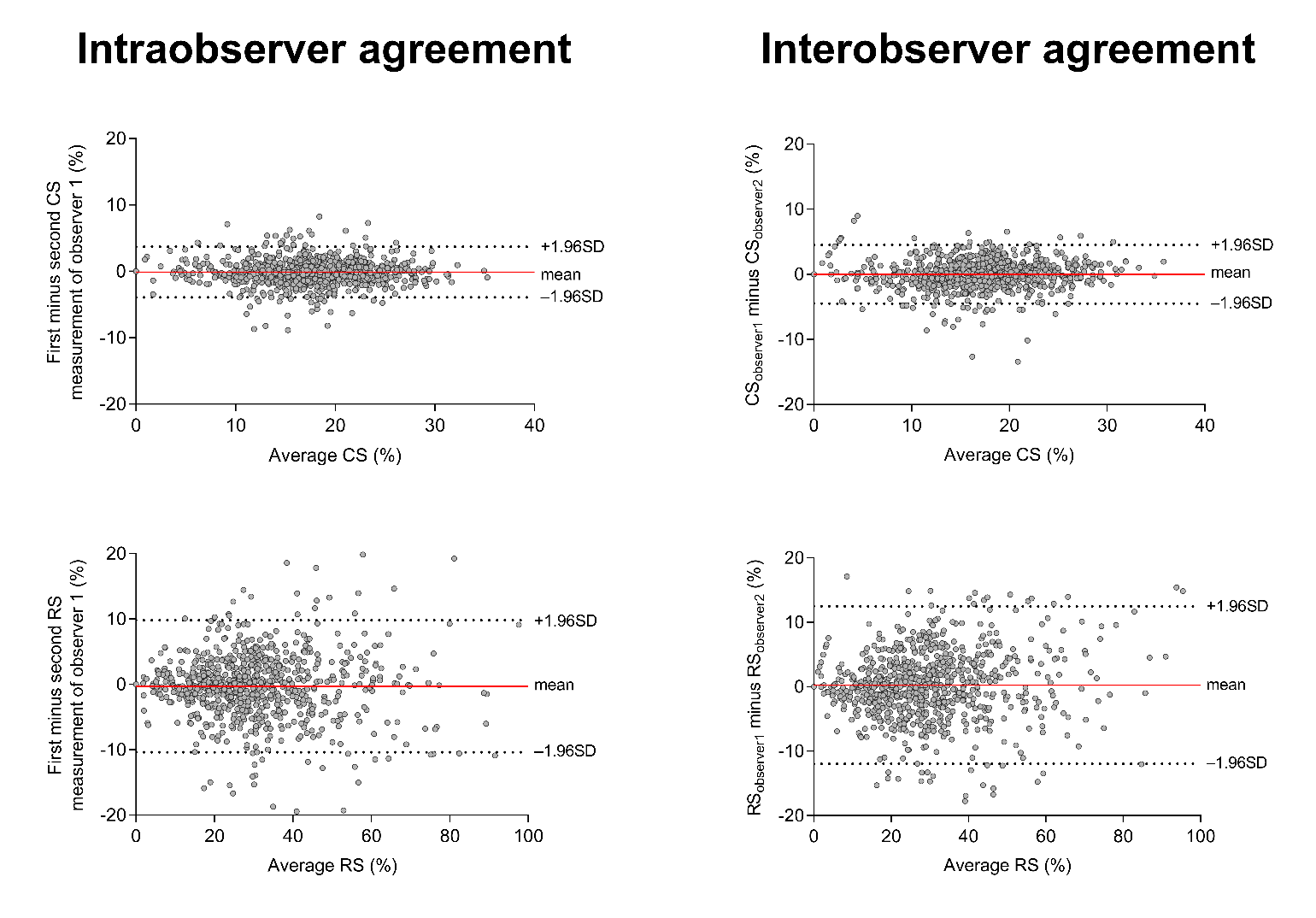


Bland-Altman plots demonstrating the intra- and interobserver agreement of segmental CS and RS. The solid red lines indicate the mean bias and the dashed black lines indicate the limits of agreement. CS = circumferential shortening; RS = radial shortening.

# Supplemental Figure 3: Relationship between global strain, LV ejection fraction and scar


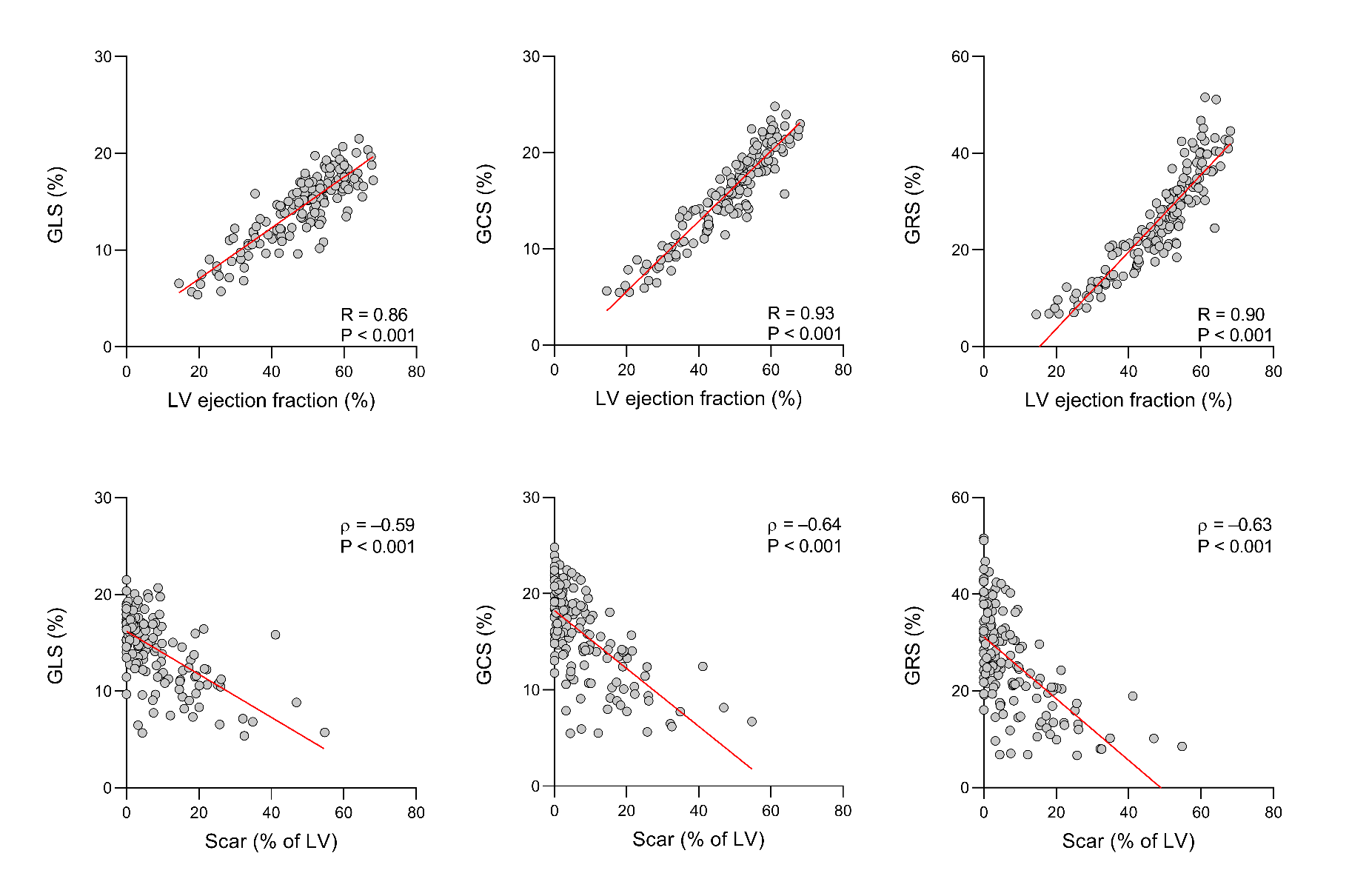


Scatterplots demonstrating the relationship of global strain parameters with LV ejection fraction (top) and scar (bottom). GLS (left), GCS (center) and GRS (right) correlate strongly with LV ejection fraction and significantly with scar. GCS = global circumferential shortening; GLS = global longitudinal shortening; GRS = global radial shortening; LV = left ventricle

# Supplemental Figure 4: Relationship between regional strain and scar


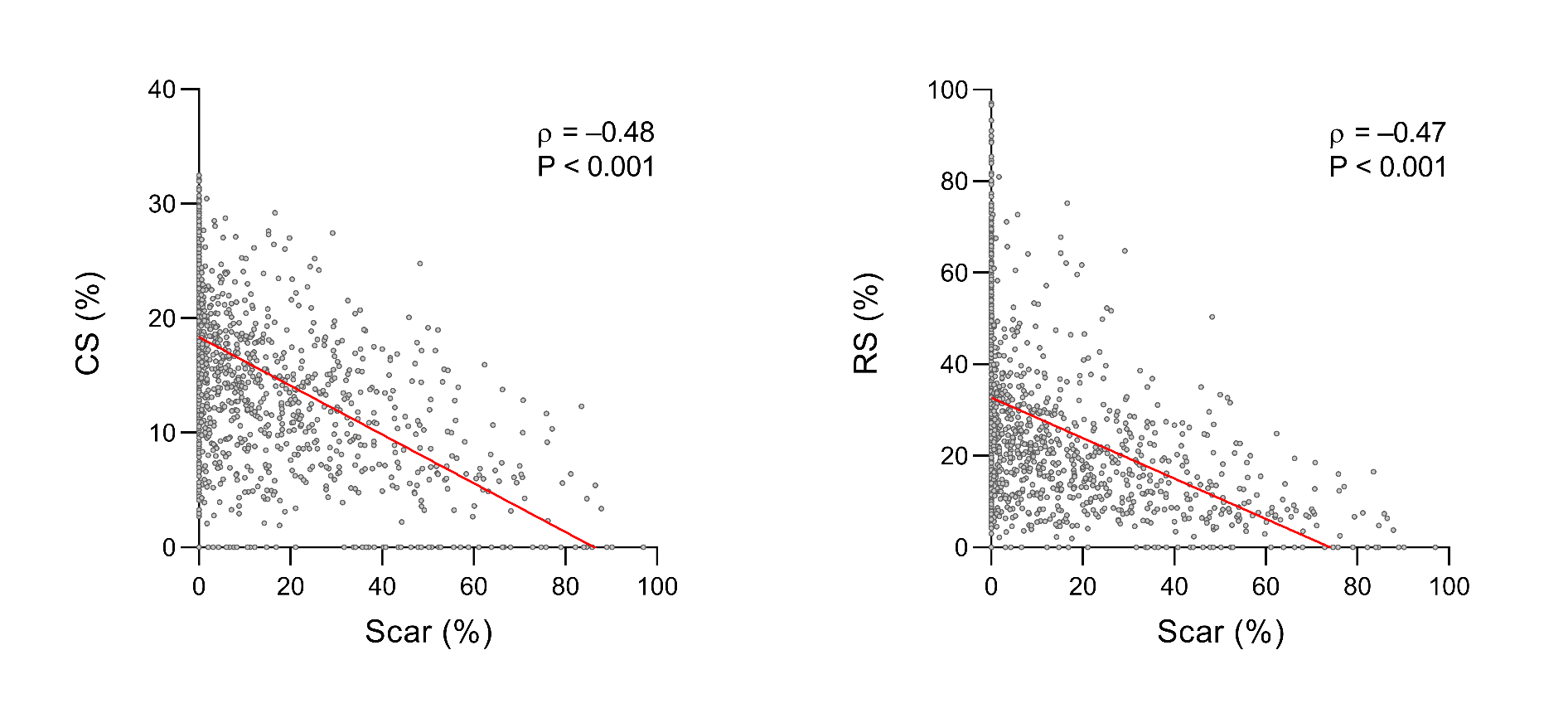


Scatterplots demonstrating the relationship of CS (left) and RS (right) with the percentage of scar on a segmental level. CS = circumferential shortening; RS = radial shortening.

# Supplemental Table 1: Global LV function in healthy volunteers (n = 100)

| Variables | Mean ± SD | 95% CI | Minimum | Maximum |
| --- | --- | --- | --- | --- |
| EDM (g)  EDV (mL)  ESV (mL)  SV (mL)  Ejection fraction (%)  GLS (%)  GCS (%)  GRS (%) | 103 ± 25  159 ± 33  65 ± 18  95 ± 20  59.7 ± 5.4  17.6 ± 1.8  18.5 ± 2.2  31.3 ± 6.1 | 59 to 146  107 to 217  39 to 99  64 to 130  51.2 to 68.4  14.4 to 20.8  15.0 to 22.5  22.1 to 43.7 | 54  88  35  48  47.9  13.3  13.6  20.8 | 156  258  108  155  69.3  21.7  23.3  46.1 |

EDM = end-diastolic mass; EDV = end-diastolic volume; ESV = end-systolic volume; GLS = global longitudinal shortening; GCS = global circumferential shortening; GRS = global radial shortening; LV = left ventricle; SV = stroke volume.
